# The expression of platelet serotonin transporter (SERT) in human obesity

**DOI:** 10.1186/1471-2202-14-128

**Published:** 2013-10-18

**Authors:** Gino Giannaccini, Laura Betti, Lionella Palego, Alessandro Marsili, Ferruccio Santini, Caterina Pelosini, Laura Fabbrini, Lara Schmid, Laura Giusti, Margherita Maffei, Mario Lanza, Mario Cristofaro, Stefano Baroni, Mauro Mauri, Paolo Vitti, Paola Fierabracci, Antonio Lucacchini

**Affiliations:** 1 Department of Pharmacy, University of Pisa, via Bonanno 6, Pisa 56126-I, Italy; 2 Department of Clinical and Experimental Medicine, University of Pisa, Via Savi 10, 56126-I, Pisa, Italy; 3 Clinical Pharmacology Unit, University Hospital “Santa Chiara”, Via Savi 10, 56126-I, Pisa, Italy; 4 Insitute of "Fisiologia Clinica", CNR, Via G. Moruzzi 1, 56124, Pisa, Italy; 5 Endocrinology Unit, University Hospital of Cisanello, via Paradisa 2, Pisa, Italy

**Keywords:** Human obesity, SERT expression, [^3^H]-paroxetine binding, Platelets

## Abstract

**Background:**

Serotonin (5-HT) is a well-known modulator of eating behavior. However, the molecular mechanisms linking its action to body weight balance have been only partially elucidated. Since platelets are a suitable peripheral model to study 5-HT transport, metabolism and release, we herein evaluated the expression of the platelet 5-HT re-uptake system (SERT) by [^3^H]-paroxetine binding assay. A cohort of 114 unrelated individuals (34 males, 80 females; age, mean ± SD: 38.57 ± 12.47 years) without major psychiatric disorders, was recruited following a naturalistic design regarding age or gender and classified accordingly to their body mass index (BMI). Subjects were divided into 5 groups: normal-weight (NW), overweight (OW) and grade I-III obese (OB) individuals. For gender analyses, data were transformed into [^3^H]-paroxetine density (B_max_)/BMI ratios to overcome both the disparity of women vs. men number and anthropometric differences between sexes.

**Results:**

[^3^H]-paroxetine B_max_ (SERT density, fmol/mg proteins) was reduced in platelet membranes of grade II (p < 0.01) and III (p < 0.001) obese subjects vs. controls and in overweight subjects (p < 0.05) vs. grade III obese individuals. Considering all patients together, a strong negative correlation between B_max_ and BMI (r = −0.449; P < 0.0001) was demonstrated. Conversely, [^3^H]-paroxetine K_D_ (dissociation constant, nM) did not differ among groups. No gender-related variation concerning B_max_/BMI ratios was observed in this cohort of subjects.

**Conclusions:**

The down-regulation of SERT in platelet membranes of severe human obesity (BMI > 35 Kg/m^2^) confirms the involvement of 5-HT system in body weight gain. Moreover, this findings may help to elucidate those monoamine-endocrine networks acting on fat storage, adipocyte signaling and energy balance. Targeting 5-HT/5-HT-related markers will possibly uncover the existence of human obesity subtypes.

## Background

Among neurotransmitters linked to appetite control, serotonin (5-HT) has a particular role: this endogenous amine is tightly involved in the regulation of feeding behavior at hypothalamic level, acting within the ventromedial and lateral nuclei [1-3]. In fact, the activity of 5-HTergic raphe and hypothalamic neurons is influenced by meal macronutrient composition and insulin secretion, as suggested by the findings that the tryptophan/large neutral amino acids concentration ratio (Trp:LNAAs) in plasma (an index of Trp availability to brain uptake) and 5-HT synthesis are both increased after a carbohydrate-rich meal [4-8]. The same concept applies to protein-rich meals or meals containing proteins with high tryptophan content (e.g. α-lactalbumin) [9], demonstrating the impact of diet upon tryptophan uptake, 5-HT production and synaptic release. On the other side, glucocorticoid response influences monoamine/5-HT transmission and receptor function in the central nervous system (CNS), thus affecting feeding behavior and macronutrient choice [10-13]. These observations clearly suggest a link between stress-response, 5-HT function, weight gain and obesity. Several studies indicate that obesity has, in most cases, a polygenic background [14-16]. Among others, genes coding for proteins involved in 5-HT system such as the 5-HT transporter (SERT or 5-HT-T) [17-21], carriers for neutral amino acids (including tryptophan) [22] and 5-HT receptor subtypes [23-28] appear to be functionally relevant in either animal or human obesity. From diet studies conducted in rodents and humans, the interest at targeting specific 5-HT sites and, in particular, SERT [29,30] is strongly increased. Structurally, SERT is a glycoprotein belonging to the super-family of membrane-bound NaCl-dependent neurotransmitter transporters, characterized by 12 putative membrane spanning domains: it promotes 5-HT clearance (re-uptake) from the extracellular milieu and modifies the sensitization state of 5-HT receptors within the nervous system or non-neural districts (gut, platelets, lymphomonocytes) [31,32]. It is a pharmacologically active site, the target of re-uptake inhibitors as tricyclic antidepressants (TCA) and Selective Serotonin Reuptake Inhibitors (SSRIs) or 5-HT releasers like fenfluramine and 3,4-methylenedioxy-*N*-methamphetamine (MDMA) [33]. Both SERT expression and 5-HT uptake function are finely tuned by protein-kinases activities and gene transcription which control, following cell necessities, conformational changes of the membrane-bound SERT protein and/or the degree of SERT partition between cytoskeleton and plasma membrane [34-37].

Data on SERT expression/affinity in peripheral districts of overweight/obese subjects are currently not available. Platelets are a valuable peripheral model that mimics 5-HT transport, metabolism and release in the CNS, since they have been characterized for many years as a surrogate of impaired 5-HT activity in subjects with psychiatric disorders, eating behavior and ageing [38-44]. Therefore, the present study aimed to evaluate human platelets SERT number or affinity according to different categories of body mass index (BMI) or genders.

## Results

### Subjects’ groups

As shown in Table 1, 114 individuals were recruited in the study and divided into 5 main BMI groups: 28 normal weight subjects (NWs), 18 overweight (OWs), 17 class I obese (OB-Is), 19 class II obese (OB-IIs) and 32 class III obese (OB-IIIs) individuals. ANOVA analysis showed a significant difference among BMIs of the groups (p < 0.0001), without noticeable age variations (p > 0.05).

**Table 1:** The 5 groups of BMIs

	**Controls *(NWs, n = 28)***	**Overweight *(OWs, n = 18)***	**Obese I *(OB-Is, n = 17)***	**Obese II *(OB-IIs, n = 19)***	**Obese III *(OB-IIIs, n = 32)***
**Age (y)**	35.11 ± 2.24	39.42± 3.10	39.76 ± 3.22	36.16 ± 2.92	41.94 ± 2.06
	(20.0-61.0)	(21.0-59.0)	(16.0-63.0)	(20.0-61.0)	(22.0-59.0)
**BMI (Kg/m**^**2**^**)**	21.39 ± 0.39	27.07 ± 0.26	32.67 ± 0.30	37.52 ± 0.34	46.34 ± 0.67
	(18.3-25)	(***) (25.4-28.8)	(***) (30.1-34.7)	(***) (35.5-39.8)	(***) (40.0-54.8)

Data are presented as mean ± S.E.M.; in parenthesis sample ranges, minimum and maximum values are shown.

ANOVA BMI and *post-hoc* Bonferroni test: (***): p < 0.001, NWs vs. OWs, OB-I/II/IIIs.

### [^3^H]-paroxetine binding experiments

**Figure 1:**
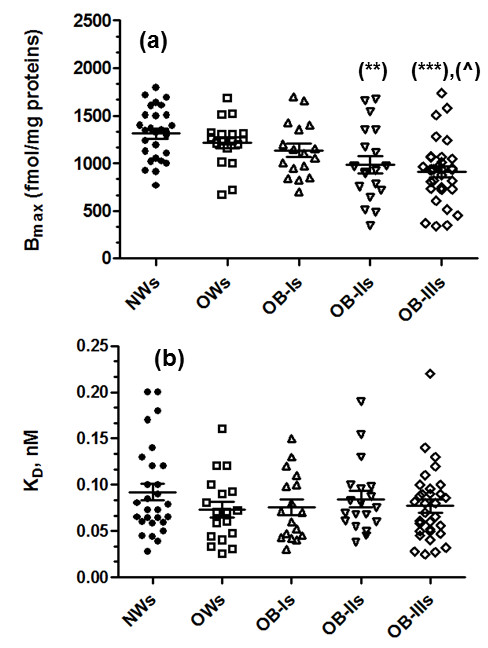
**SERT parameters and ANOVA analysis.** Scattergram plots of **a)** [^3^H]-paroxetine B_max_, fmol/mg protein (SERT number) and **b)** [^3^H]-paroxetine K_D_, nM (SERT affinity), obtained in platelets from individuals of the 5 BMI (Kg/m^2^) groups. Among group ANOVA: p < 0.0001 (all groups); Bonferroni *post-hoc* tests showed significant tests: (**): OB-IIs vs. NWs; (***): OB-IIIs vs. NWs and (^): OB-III vs. OWs. Each scattergram plot also shows the mean ± SEM.

Equilibrium saturation and Scatchard analysis of [^3^H]-paroxetine specific binding showed a single population of high-affinity recognition sites in platelet membranes from all the subjects under investigation, clearly indicating the labeling of a single protein. The specific binding was about 90% of total binding at the K_D_ concentration. The [^3^H]-paroxetine B_max_ (fmoles/mg protein) values, corresponding to SERT expression in platelet membranes, were: 1311 ± 51.29 (min.-max: 767–1795) in NWs; 1215 ± 59.44 (min.-max: 665–1685) in OWs; 1137 ± 70.36 (min.-max: 700-1700) in OB-Is; 986.4 ± 89.73 (min.-max.: 344–1675) in OB-IIs; 906.8 ± 58.51 (min.-max: 336–1737) in OB-IIIs. The [^3^H]-paroxetine K_D_ values (nM), corresponding to the SERT protein affinity state for the specific ligand, were: 0.092 ± 0.009 (min.-max: 0.028-0.20) in NWs; 0.073 ± 0.0085 (min.-max: 0.025-0.16) in OWs; 0.076 ± 0.009 (min.-max: 0.03-0.15) in OB-Is; 0.085 ± 0.009 (min.-max: 0.038-0.19) in OB-IIs; 0.077± 0.007 (min.-max: 0.025-0.22) in OB-IIIs. Individual results for [^3^H]-paroxetine B_max_ and K_D_, obtained from the 5 BMI groups of subjects, are reported in Figure 1(a,b). ANOVA analysis showed a significant difference between the [^3^H]-paroxetine B_max_ means of the 5 BMI groups (p < 0.0001); after the *post-hoc* Bonferroni correction test, B_max_ mean values were significantly reduced in OB subjects class II-III (BMI > 35 kg/m^2^) vs. NWs (p < 0.01 and p < 0.001, respectively) (Figure 1a); B_max_ values were also decreased in OB-IIIs respect to OWs (p < 0.05) (Figure 1a).

### Correlation analyses and gender impact

**Figure 2:**
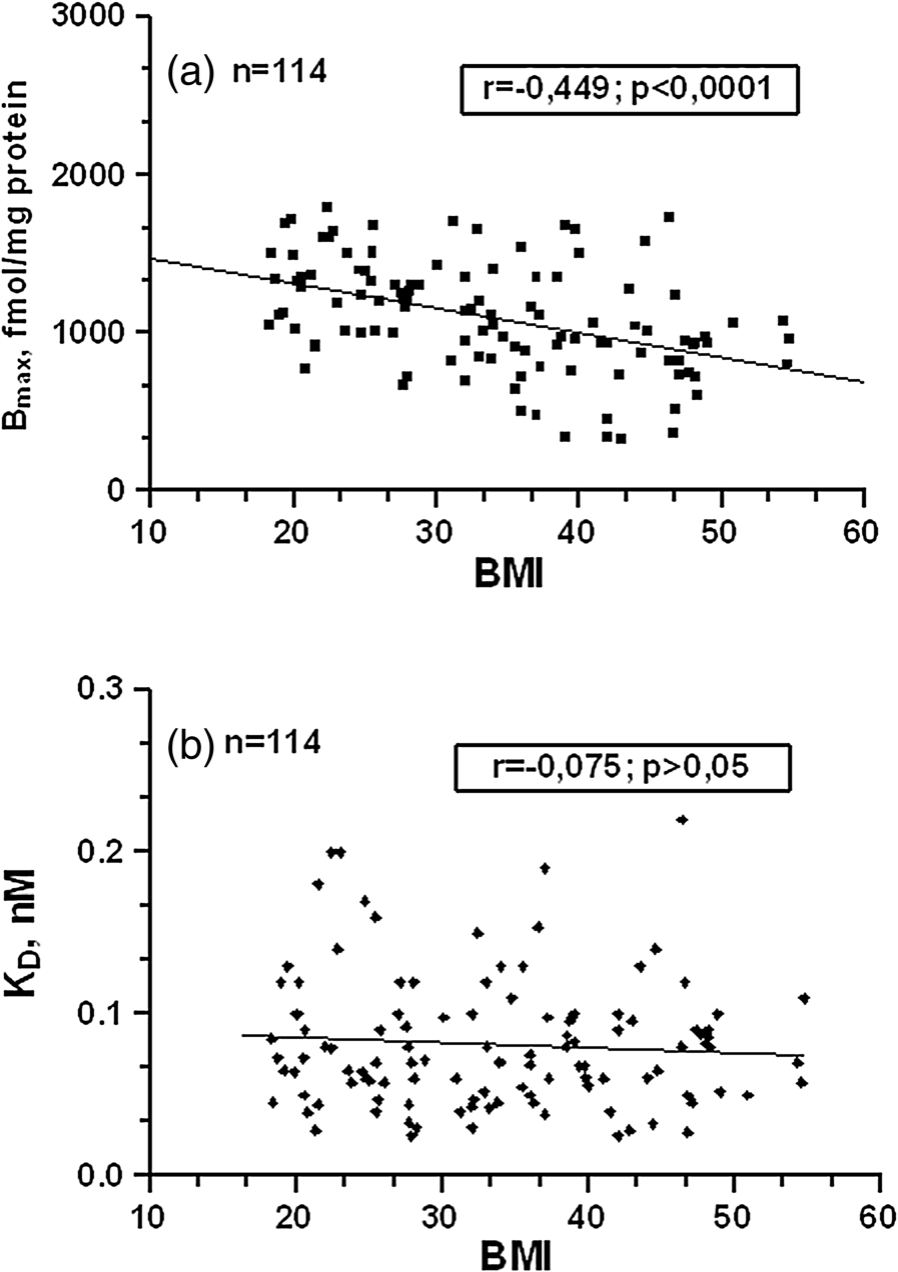
**Correlations of platelet SERT parameters with subjects’ BMI (Kg/m**^**2**^**).** Correlations between **a)** [^3^H]-paroxetine B_max_ (fmol/mg protein), **b)** [^3^H]-paroxetine K_D_ (nM) and BMI. Panels inside figures report the corresponding Pearson r coefficient and its statistical significance. Lines in **a)** and **b)** represents data linear fit from linear regression analysis.

**Figure 3:**
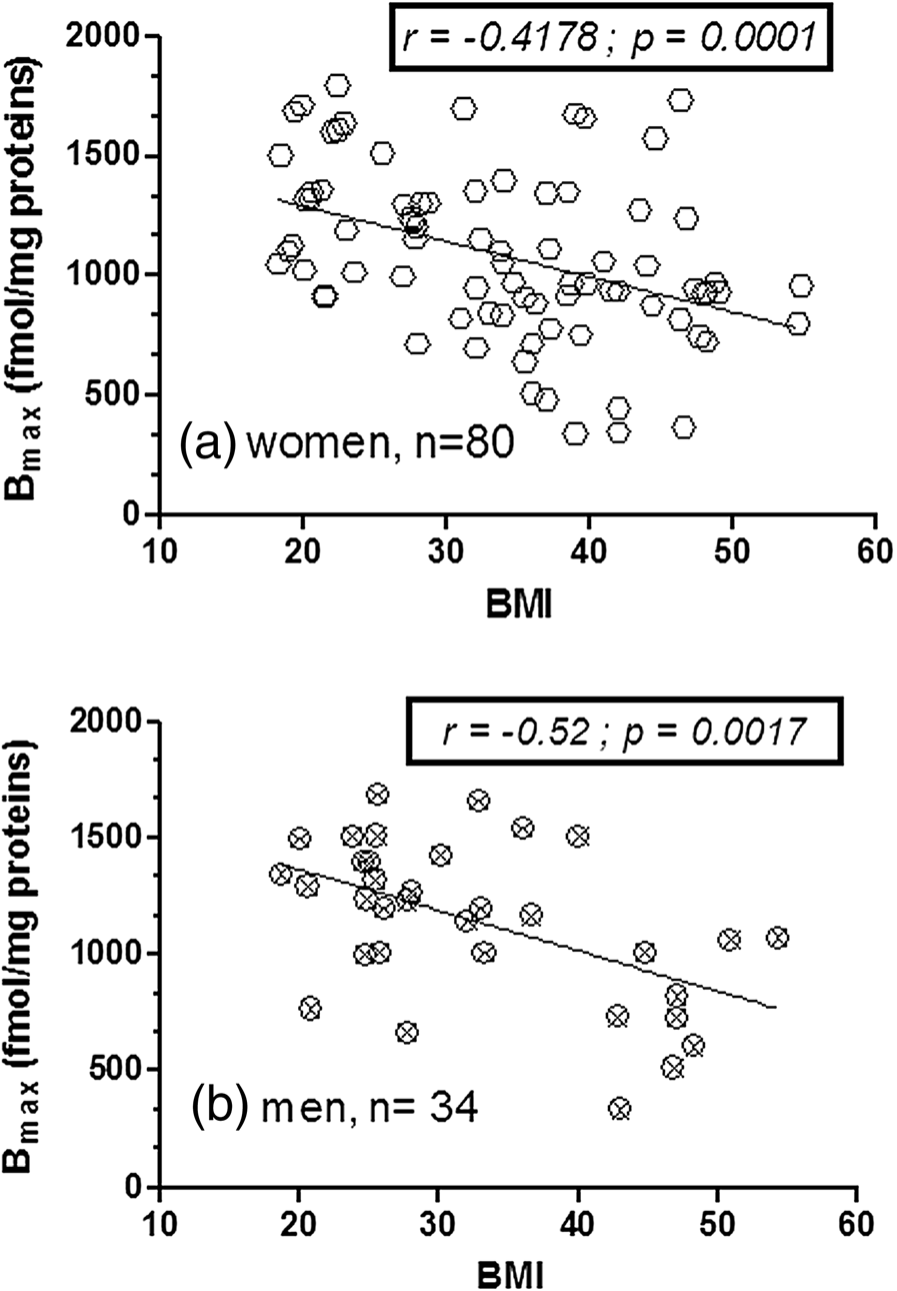
**Correlations of platelet SERT density with BMI (Kg/m**^**2**^**) by gender.** Correlation analysis between [^3^H]-paroxetine B_max_ (fmol/mg protein) with BMI in **a)** women (n=80) and **b)** men (n=34). Panels inside figures report the Pearson r coefficient and its statistical significance. Lines in **a)** and **b)** represents data linear fit from linear regression analysis.

**Figure 4:**
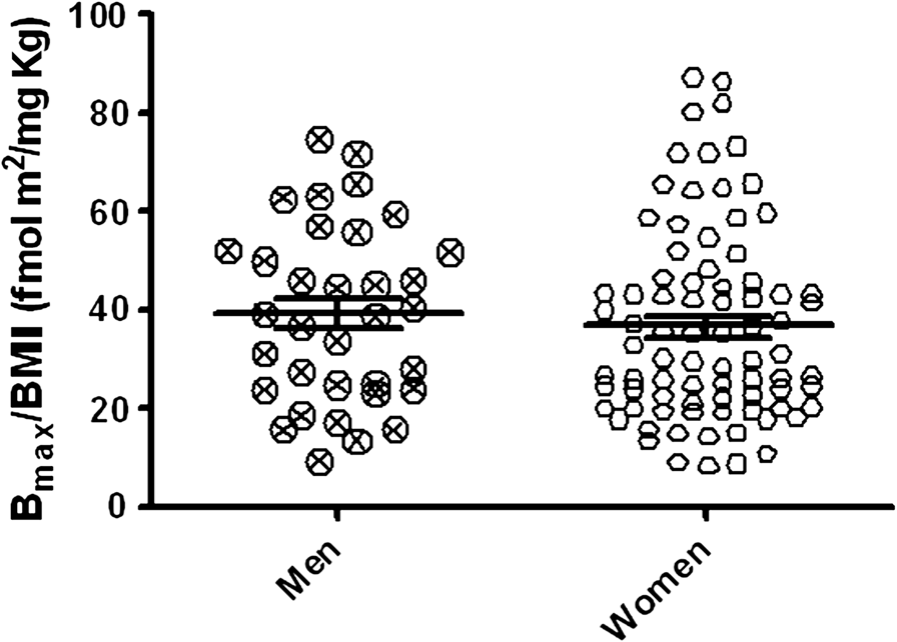
**Comparison of [**^**3**^**H]-paroxetine B**_**max**_**/BMI ratios (SERT density/BMI, fmol m**^**2**^**/mg Kg), in males and females.** Scattergram plots of [^3^H]-paroxetine B_max_/BMI ratios obtained in men and women. Each scattergram plot also shows the mean ± SEM.

Among-groups differences in SERT expression were additionally sustained by the significant negative correlation between [^3^H]-paroxetine B_max_ and BMI both in the whole cohort (r: -0.449, p < 0.0001; Figure 2a) and by gender sub-analysis in women (r = −0.4178 ; p = 0.0001; Figure 3a) and men ( r = −0.52 ; p = 0.0017; Figure 3b). No significant gender related differences in subjects’ variables (Table 2), as well as in B_max_/BMI ratio (fmol m^2^ /mg Kg) were found (p *= ns*), (Figure 4). The B_max_/BMI ratio was: 39.12 ± 3.11 (min.-max: 9.33-74.80) in men and 36.53 ± 2.21 (min.-max:7.92-87.06) in women. No significant variation was reported in SERT affinity (K_D_) among the BMI based groups (Figures 1b and 2b).

**Table 2:** Gender effect on subject’s variables

	***Men, n = 34***	***Women, n = 80***
**Age (years)**	37.71 ± 2.28	38.94 ± 1.36
	(20.00-63.00)	(15.00-61.00)
**BMI (kg/m**^**2**^**)**	32.74 ± 1.74	34.05 ± 1.11
	(18.71-54.30)	(18.30-54.80)
**[**^**3**^**H]-paroxetine B**_**max **_**(fmol/mg protein)**	1142 ± 58.31	1085 ± 39.02
	(336–1685)	(344–1795)
**[**^**3**^**H]-paroxetine K**_**D **_**(nM)**	0.071 ± 0.006	0.085 ± 0.005
	(0.028-0.170)	(0.025-0.220)

Data are presented as mean ± S.E.M. and ranges (minimum and maximum values).

## Discussion

Serotonin (5-HT), primarily produced in CNS raphe nuclei and gut, plays a wide-ranging modulatory role at the level of several homeostatic responses. In particular, CNS 5-HT regulates many amongst the main individual adaptive-relational abilities to react to environmental changes, such as feeding behavior, thermoregulation, motor activity, libido, cognition, impulsivity, aggressiveness, nociception and mood. Besides, 5-HT also acts on peripheral tissues and organs, modulating the immune and flogistic responses, as well as blood stem cells differentiation, hemodynamic function and intestinal peristalsis [45]. Despite 5-HT has been extensively studied in recent years, the link between the expression of 5-HT transporter (SERT), the pivotal protein regulating its extra- and intra-cell concentrations, and human obesity has been supported by few studies. By single-photon emission tomography (SPECT) analysis in midbrain areas of obese women affected by binge eating disorder (BED), a reduction in SERT density has been reported [46], and this reduction was rescued by SSRI therapy [47].

A more recent *in vivo* PET study, using a iodinate tracer ([^123^I]-nor-β-CIT) in midbrain areas of monozygotic twins, has shown a higher SERT density in co-twins with higher BMI [48]. The latter study was conducted in the Finnish population, (presenting a reduced genetic variance than other human ethnic groups) and selected twins were prevalently women.

Conversely, other PET investigations on unrelated healthy volunteers using a different SERT ligand ([^11^C]-DASB), have shown a negative correlation between cerebral SERT expression and BMI [49,50]. Our study clearly demonstrates a reduced SERT number in platelet membranes of severely obese subjects (> 35 kg/m^2^) and a negative correlation between platelet SERT B_max_ and BMI in human obesity. Instead, the lack of significant changes in the SERT affinity parameter K_D_ suggests a comparable SERT protein conformation in lean and obese individuals. All these studies substantiate the link between 5-HT activity, SERT expression and weight gain, but discrepancies are present. An explanation of this discrepancy can be found putting all these data in the context of SERT regulatory pathways.

As introduced before, protein SERT expression is a model of “fine-tuned” regulation of membrane-bound proteins. Beside undergoing a short-term up and down-regulation, SERT presence in cell membranes can be long-term modulated through positive and negative signals, allowing long-lasting cell adaptation to the extracellular content of 5-HT or other related stimuli. The balance between the converging short and long-term regulatory pathways of SERT defines its expression and affinity states during developmental stages, under physiological and pathological conditions.

We have previously shown that SERT protein expression in platelets (in plasma membrane and intracellular pools) is regulated by megakaryoblast cell differentiation processes [51]. We have also reported an up-regulated translocator protein TSPO expression in discrete brain regions of *ob/ob* mice, without appreciable changes in SERT number either in the brain or in platelets [52]. Since leptin has been found to down-regulate SERT expression [53], we hypothesized that *ob/ob* animals, during their development, can modulate SERT expression through the activation of alternative regulatory pathways, without excluding modified SERT reserve and 5-HT responsiveness. In the present study, a reduced platelet SERT in severe obese **s**ubjects (grade II and III) has been shown. This finding mirrors at the peripheral levels what previously reported in the brain [50]. In contrast to mutant leptin-lacking *ob/ob* mice, a link between human obesity, often associated with high serum leptin [54,55] and SERT regulatory cascades leading to its reduction or internalization can be hypothesized. The implications of regulatory mechanisms on reduced SERT expression in obesity is indirectly supported by studies conducted on double knockout SERT(−/−)/brain derived neurotrophic factor (BDNF) (+/−) mice [56,57] revealing the regulatory role of either other monoamine protein markers or trophic factors on 5-HT physiology and activity on body weight balance. Nevertheless, currently, a clear explanation for the lower SERT expression found in platelets of severe obese individuals is lacking. Platelet 5-HT can be part of a network involving adipokines, cytokines and inflammatory responses [58]. This is supported by the report of adipocytes expressing 5-HT receptor subtypes [59] and, more recently, even SERT [60], suggesting that adipose tissue and 5-HT system interact with each other. It is possible that the reduced SERT expression is due to impaired 5-HT synthesis and activity in obese subjects [3,61], as reported for neurotic behaviors and personality traits, and that altered SERT/5-HT receptors and/or SERT regulation underscore obesity. In this study, none of the recruited subjects had a present or past history of a major psychiatric disorder, but some of them could present personality traits that could be possibly linked to susceptibility to obesity [57,62]. On the other side, imbalanced appetite hormones, adipokines or gut hormones could counter-regulate SERT expression.

The controversy between reduced SERT expression in obese subjects and increased midbrain SERT in acquired obesity, as reported in monozygotic co-twins with a higher BMI [48], can be explained by different SERT regulatory processes during gene-environment interactions. Specifically, the selection criteria applied in the Finnish study could have included higher BMI co-twins under particular lifestyles and/or changes of dietary habits leading to SERT up-regulation, as observed in rodent models of acquired obesity [63]. At the same time, considering the experimental design of the Finnish study, selected twins could also bear a genotype linked to vulnerability to stress as SERT-reducing obese subjects [64]. Moreover, of note, our investigation and that by Erritzoe et al. (2011) [50] much differ from the Finnish study [48] in terms of: a) evaluated BMI ranges; b) employed technical procedures (e.g., PET vs. *in vitro* binding experiments carried in membranes; different SERT binding tracers); c) sample size of recruited subjects.

Despite the well-known gender-related differences in obesity and fat distribution, we did not found appreciable differences in B_max_/BMI ratios in males vs. females, suggesting a gender-independent effect of BMI on SERT expression in platelets of severe obese individuals.

## Conclusions

Analyzing the biggest cohort of the literature so far, our study demonstrates, for the first time, that SERT density is reduced in plasma membranes of circulating platelets of severe (class II/III, BMI > 35 kg/m^2^) obese subjects, without gender-related differences. Nevertheless, the complexity of SERT regulation needs to be investigated further. A multivariate statistical elaboration in normal, overweight and obese subjects is currently in progress in order to better define the contribution of energy metabolism/adipocyte function on the modulation of platelet SERT (number and function) in obese individuals. Moreover, we suggest to better evaluate the role of 5-HT in body weight balance through the measure of other parameters such as 5-HT re-uptake function, intra-platelet/bloodstream 5-HT levels, intra-platelet SERT content, plasma large-neutral amino acids, BDNF, TSPO as well as the binding and sensitization state of 5-HT receptor subtypes in obese subjects. Microarray gene, peptide/protein analyses and metabolomics would be helpful to identify involved signals, effectors and regulatory cascades, also in other SERT expressing districts such as the gut or adipose tissue. The targeting of 5-HT-related gene/proteins and other monoamine or endocrine biomarkers would help to detect different subtypes of human obesity, possibly triggered by distinct biological causes, allowing the development of novel therapeutic strategies.

## Methods

### Chemicals

[^3^H]-paroxetine (specific activity: 15.5 Ci/mmol) was purchased from Perkin-Elmer, Life Science, Milan, Italy. All other reagents were of the best analytical grade.

### Subjects

One hundred and fourteen (Italian) subjects (34 M; 80 W; age: 38.57 ± 12.47 years) with a BMI ranging between 18.30 and 54.80 Kg/m^2^ (33.54 ± 9.923 Kg/m^2^) were enrolled for the present study. Normal weight subjects were recruited from the medical and laboratory staff of the Endocrinology Center. Overweight and obese (BMI > 25 Kg/m^2^) subjects were recruited among the patients of the Obesity Center, Endocrinology Unit 1, University of Pisa. Exclusion criteria were: active cancer, heart, liver or kidney diseases; presence of hematological or neurological illnesses, a positive history for substance abuse and psychiatric (Axis I) disorders assessed by Structured Clinical Interview for DSM-IV Axis-I diseases (SCID/I diagnostic criteria).

Subjects assuming substances acting on SERT, other psychotropic agents or estro-progestinic drugs and Non-Steroidal Anti-Inflammatory Drugs (NSAIDs) were admitted to the study after a 3 months and 10 long days withdrawal, respectively. Assuming hypotensive or interfering with carbohydrate-lipid metabolism (insulin, oral hypoglycemic compounds, statines) drugs was an exclusion criteria as well.

Height was measured, while subjects were standing, using standardized techniques and equipment. Body weight was measured by a precision instrument and electronic scale (± 0.1 Kg). A regular informed consent approved by the Ethics Committee of the Pisa University was signed by all subjects after reading a full explanation of the project.

### Platelet sampling

To avoid catecholamine release as well as circadian rhythm interference, peripheral venous blood (30 ml) was drawn from fasting subjects in clinostat position between 8.30 and 10 a.m. Blood was collected into plastic tubes containing 5 ml of anticoagulant (2.2% sodium citrate, 1.2% citric acid) and centrifuged at low-speed (150 g) for 15 min at 23°C to separate the platelet rich plasma (PRP). Platelets were then precipitated from PRP by an ensuing centrifugation at 1,500 g for 15 min at 23°C and counted automatically with a flux cytometer (Cell-dyn 3500 system; Abbott, Milano, Italy). Platelets were then washed by centrifugation for 10 min at 10,000 g, 4°C and resulting pellets stored at −80°C until assay, performed within 1 week.

### Platelet membrane preparation

At the time of the assay, platelets were re-suspended in 10 volumes (*w:v*) ice-cold 5 mM Tris–HCl buffer (pH 7.4) containing 5 mM EDTA and protease inhibitors (benzamidine 160 μg/ml, bacitracine 200 μg/ml; trypsine soy inhibitor 20 μg/ml). After homogenization by Ultraturrax, samples were centrifuged at 48,000 g for 15 minutes at 4°C. The ensuing pellets were washed twice in 10 volumes (*w:v*) ice-cold 50 mM Tris–HCl buffer (pH 7.4) by a centrifugation step, as above indicated. The final membrane pellets were suspended in the assay buffer consisting in a 50 mM Tris–HCl buffer (pH 7.4), containing 120 mM NaCl and 5 mM KCl. Protein content was determined by the Bradford’s method (Bio-rad), using γ-globulins as the standard.

### [^3^H]-Paroxetine binding assay

SERT binding parameters (maximal binding capacity, B_max_, fmol/mg protein; dissociation constant, K_D_, nM) were evaluated in platelet membranes by measuring the specific binding of [^3^H]-paroxetine. The [^3^H]-paroxetine B_max_ represents the specific density (number) or the degree of SERT protein expression on platelet membranes of each enrolled subject, while K_D_ being the main index of ligand-to-protein affinity. Saturation experiments were conducted as follows: 100 μl of membranes (corresponding to 50–100 μg proteins/tube) were incubated in assay buffer (50 mM Tris–HCl, 5 mM KCl, 120 mM NaCl, pH 7.4) with five increasing concentrations of [^3^H]-paroxetine (0.08-1.5 nM) in a final assay volume of 2 ml. Non-specific binding was performed, for each [^3^H]-paroxetine concentration point, in the presence of 10 μM fluoxetine, as cold displacer. Incubation was performed at 22-24°C for 60 min and halted by rapid filtration using Whatman GF/C glass fiber filters in a Brandell filtration apparatus. Filters were then washed three times with 5 ml ice-cold buffer assay, put into pony vials and measured for radioactivity (dpm) through a liquid phase scintillation β-counter Packard 1600 TR. Specific binding was obtained by subtracting residual binding in the presence of 10 μM fluoxetine from total binding.

### Data analysis

For statistical analyses, the subjects were divided into groups according to BMI classes: normal-weight (NW, controls), overweight (OW) and grade I-III obese (OB) individuals [65]. Equilibrium-saturation binding data, maximum binding capacity (Bmax, fmol/mg of protein) and dissociation constant (K_D_, nM), were calculated by the iterative curve-fitting computer programs EBDA-LIGAND (Kell for Windows, v. 6.0) [66] and Graph-Pad Prism (version 3 and 5, San Diego, CA, USA). Results of descriptive statistical analyses were reported as the mean ± the Standard Error of the Mean (S.E.M.), when not differently indicated in the text. For inferential analyses relating SERT expression and affinity with obesity ANOVA followed by the Bonferroni *post-hoc* test as well as *t*-test Student for unpaired data (gender influence) were used; Pearson correlations analyses and linear regression tests between platelet [^3^H]-paroxetine binding parameters and BMI values were also performed in men and women separately between platelet [^3^H]-paroxetine binding parameters and BMI values were also performed in men and women separately. For gender-specific *t-*test analysis, binding densities were normalized for BMI in women and men separately, obtaining B_max_/BMI values (fmol × m^2^ height squared/mg proteins × Kg body weight). For all statistical analyses, Graph-Pad Prism software was used and the significance threshold was set at p = 0.05.

## Competing interests

The authors declare that they have no competing interests.

## Authors’ contributions

GG and AL conceived the study, participated to its design and coordinated all the study steps; FS, MM and PV participated to the study development while FS, AM, MMr and PF recruited and clinically evaluated the subjects; LB, LF, LS and ML conducted binding assays; CP and SB were responsible of blood sampling and processing; LP, LF, LG and MC elaborated experimental results; LP, LB and AM wrote the manuscript. All authors read and approved the final manuscript.

## References

[B1] Leibowitz SF Alexander JT Hypothalamic serotonin in control of eating behaviour, meal size and body weight Biol Psychiatry 1989 14 851 864 10.1016/s0006-3223(98)00186-3 9807640

[B2] Schwartz DH Hernandez L Hoebel BG Serotonin release in lateral and ventral hypothalamus during feeding and its anticipation Brain Res Bull 1990 14 797 802 10.1016/0361-9230(90)90173-W 2289169

[B3] Blundell JE Serotonin and the biology of feeding Am J Clin Nutr 1992 14 1555 1595 10.1093/ajcn/55.1.155s 1728826

[B4] Gessa GL Biggio G Fadda F Corsini GU Tagliamonte A Effect of the oral administration of tryptophan-free amino acid mixtures on serum tryptophan, brain tryptophan and serotonin metabolism J Neurochem 1974 14 869 870 10.1111/j.1471-4159.1974.tb04308.x 4407107

[B5] Caballero B Finer N Wurtman RJ Plasma amino acids and insulin levels in obesity: response to carbohydrate intake and tryptophan supplements Metab 1988 14 672 676 10.1016/0026-0495(88)90089-3 3290625

[B6] Benton D Carbohydrate ingestion, blood glucose and mood Neur Biobehav Rev 2002 14 293 308 10.1016/S0149-7634(02)00004-0 12034132

[B7] Kendzor D Appelhans B Hedeker D Pagoto S Abuse potential of carbohydrates for overweight carbohydrate cravers Psychopharmacology (Berl) 2008 14 637 647 10.1007/s00213-008-1085-z 18273603 PMC2829437

[B8] Wurtman RJ Non-nutritional uses of nutrients Eur J Pharmacol 2011 14 S10 S15 21816139 10.1016/j.ejphar.2011.07.005

[B9] Fernstrom JD Large neutral amino acids: dietary effects on brain neurochemistry and function Amino Acids 2012 10.1007/s00726-012-1330-y 10.1007/s00726-012-1330-y 22677921

[B10] Ely DR Dapper V Marasca J Correa JB Gamaro GD Xavier MH Effect of restraint stress on feeding behavior of rats Physiol Behav 1997 14 395 398 10.1016/S0031-9384(96)00450-7 9089758

[B11] Van de Kar LD Blair ML Forebrain pathways mediating stress-induced hormone secretion Front Neuroendocrinol 1999 14 1 48 10.1006/frne.1998.0172 9882535

[B12] Carrasco GA Van der Kar LD Neuroendocrine pharmacology of stress Eur J Pharmacol 2003 14 235 272 10.1016/S0014-2999(03)01285-8 12600714

[B13] Torres S Nowson C Relationship between stress, eating behavior and obesity Nutr 2007 14 887 894 10.1016/j.nut.2007.08.008 17869482

[B14] Barsh GS Farooqi S O’Rahilly S Genetics of body weight regulation Nat 2000 14 644 651 10.1038/35007519 10766251

[B15] Yang W Kelly T He J Genetic epidemiology of obesity Epidemiol Rev 2007 14 49 61 10.1093/epirev/mxm004 17566051

[B16] Bell CG Walley AJ Froguel P The genetics of human obesity Nature Rev Gen 2005 14 221 234 10.1038/nrg1556 15703762

[B17] Lam DD Heisler LK Serotonin and energy balance: molecular mechanisms and implications for type 2 diabetes Expert Rev Mol Med 2007 14 1 24 17316471 10.1017/S1462399407000245

[B18] Sookoian S Gemma C García SI Gianotti TF Dieuzeide G Roussos A Tonietti M Trifone L Kanevsky D González CD Pirola CJ Short allele of serotonin transporter gene promoter is a risk factor for obesity in adolescents Obesity (Silver Spring) 2007 14 271 276 10.1038/oby.2007.519 17299098

[B19] Sookoian S Gianotti TF Gemma C Burgueño A Pirola CJ Contribution of the functional 5-HTTLPR variant of the SLC6A4 gene to obesity risk in male adults Obesity (Silver Spring) 2008 14 488 491 10.1038/oby.2007.64 18239665

[B20] Fuemmeler BF Agurs-Collins TD McClernon FJ Kollins SH Kail ME Bergen AW Ashley-Koch AE Genes implicated in serotonergic and dopaminergic functioning predict BMI categories Obesity (Silver Spring) 2008 14 348 355 10.1038/oby.2007.65 18239643 PMC2919156

[B21] Garfield AS Heisler LK Pharmacological targeting of the serotonergic system for the treatment of obesity J Physiol 2009 14 Pt 1 49 60 19029184 10.1113/jphysiol.2008.164152 PMC2670022

[B22] Suviolahti E Oksanen LJ Ohman M Cantor RM Ridderstrale M Tuomi T Kaprio J Rissanen A Mustajoki P Jousilahti P Vartiainen E Silander K Kilpikari R Salomaa V Groop L Kontula K Peltonen L Pajukanta P The SLC6A14 gene shows evidence of association with obesity J Clin Invest 2003 14 1762 1772 14660752 10.1172/JCI17491 PMC281637

[B23] Tecott LH Sun LM Akana SF Strack AM Lowenstein DH Dallman MF Julius D Eating disorder and epilepsy in mice lacking 5-HT_2C_ serotonin receptors Nat 1995 14 542 546 10.1038/374542a0 7700379

[B24] Heisler LK Chu H Tecott LH Epilepsy and obesity in serotonin 5-HT2C receptor mutant mice Ann NY Acad Sci 1998 14 74 78 10.1111/j.1749-6632.1998.tb10175.x 9928241

[B25] Aubert R Betoulle D Herbeth B Siest G Fumeron F 5-HT_2A_ receptor gene polymorphism is associated with food and alcohol intake in obese people Int J Obes Relat Metab Disord 2000 14 920 924 10.1038/sj.ijo.0801253 10918541

[B26] Bouwknecht JA van der Gugten J Hijzen TH Maes RA Hen R Olivier B Male and femal 5-HT_1B_ receptor knockout mice have higher body weights than wild types Physiol Behav 2001 14 507 516 10.1016/S0031-9384(01)00589-3 11790410

[B27] Woolley ML Bentley JC Sleight AJ Mardsen CA Fone KC A role for 5-HT6 receptors in retention of spatial learning in the Morris water maze Neuropsychopharmacol 2001 14 210 219 10.1016/s0028-3908(01)00056-9 11489457

[B28] Bechtholt B Smith K Gaughan S Lucki I Sucrose intake and fasting glucose levels in 5-HT_1A_ and 5-HT_1B_ receptor mutant mice Physiol Behav 2008 14 659 665 10.1016/j.physbeh.2007.11.006 18155098 PMC2366078

[B29] Leibowitz SF Alexander JT Cheung WK Weiss GF Effects of serotonin and the serotonin blocker metergoline on meal patterns and macronutrient selection Pharmacol Biochem Behav 1993 14 185 194 10.1016/0091-3057(93)90103-Z 8516357

[B30] Corsica JA Spring BJ Carbohydrate craving: a double-blind, placebo-controlled test of the self-medication hypothesis Eat Behav 2008 14 447 454 10.1016/j.eatbeh.2008.07.004 18928908 PMC2632958

[B31] Marazziti D Rossi A Giannaccini G Baroni S Lucacchini A Cassano GB Presence and characterization of the serotonin transporter in human resting lymphocytes Neuropsychopharmacol 1998 14 154 159 10.1016/S0893-133X(97)00204-2 9629569

[B32] Iceta R Mesonero JE Aramayona JJ Alcalde A Molecular characterization and intracellular regulation of the human serotonin transporter in Caco-2 cells J Physiol Pharmacol 2006 14 119 130 16601320

[B33] Rothman RB Blough BE Baumann MH Dual DA/5-HT releasers: potential treatment agents for stimulant addiction Exp Clin Psychopharmacol 2008 14 458 474 19086767 10.1037/a0014103 PMC2683464

[B34] Zahniser NR Doolen S Chronic and acute regulation of Na+/Cl- dependent neurotransmitter transporters: drugs, substrates, presynaptic receptors, and signaling systems Pharmacol Ther 2001 14 21 55 10.1016/S0163-7258(01)00158-9 11750035

[B35] Zhu CB Hewlett WA Feoktistov I Biaggioni I Blakely RD Adenosine receptor, protein kinase G, and p38 mitogen-activated protein kinase-dependent up-regulation of serotonin transporters involves both transporter trafficking and activation Mol Pharmacol 2004 14 1462 1474 10.1124/mol.65.6.1462 15155839

[B36] Carneiro AM Blakely RD Serotonin, protein-kinase C-, and Hic-5-associated redistribution of the platelet serotonin transporter J Biol Chem 2006 14 24769 24780 10.1074/jbc.M603877200 16803896 PMC3875312

[B37] Ramamoorthy S Samuvel DJ Buck ER Rudnick G Jayanthi LD Phosphorylation of threonine residue 276 is required for acute regulation of serotonin transporter by cyclic GMP J Biol Chem 2007 14 11639 11647 17310063 10.1074/jbc.M611353200

[B38] Mellerup ET Plenge P Engelstoft M High affinity binding of [^3^H]-Paroxetine and [^3^H]-Imipramine to human platelet membranes Eur J Pharmacol 1983 14 303 309 10.1016/0014-2999(83)90321-7 6233162

[B39] Stahl SM The human platelet: A diagnostic and research tool for the study of biogenic amines in psychiatry Arch Gen Psychiatry 1997 14 509 516 10.1001/archpsyc.1977.01770170019001 140632

[B40] Aharanovitz O Granot Y Stimulation of mitogen-activated protein kinase and Na^+^/H^+^ exchanger in human platelets J Biol Chem 1996 14 16494 16499 10.1074/jbc.271.28.16494 8663100

[B41] Marazziti D Baroni S Rossi A Masala I Giannaccini G Gori V Lucacchini A Cassano GB Pharmacological characterization of the serotonin transporter in young and elderly subjects Neuropsychobiology 2001 14 78 83 10.1159/000054920 11490175

[B42] Ramacciotti CE Coli E Paoli R Marazziti D Dell’Osso L Serotonergic activity measured by platelet ^3^H-paroxetine binding in patients with eating disorders Psychiatry Res 2003 14 33 38 10.1016/S0165-1781(03)00059-3 12759159

[B43] Tardito D Mori S Racagni G Smeraldi E Zanardi R Perez J Protein kinase A activity in platelets from patients with bipolar disorder J Affect Dis 2003 14 249 253 10.1016/S0165-0327(02)00065-4 12943955

[B44] Martini C Trincavelli ML Tuscano D Carmassi C Ciapparelli A Lucacchini A Cassano GB Dell’Osso L Serotonin mediated phosphorylation of extracellular regulated kinases in platelets of patients with panic disorder versus controls Neurochem Int 2004 14 627 639 10.1016/j.neuint.2003.09.004 15016478

[B45] Mohammad-Zadeh LF Moses L Gwaltney-Brant SM Serotonin: a review J Vet Pharmacol Ther 2008 14 187 199 10.1111/j.1365-2885.2008.00944.x 18471139

[B46] Kuikka JT Tammela L Karhunen L Rissanen A Reduced serotonin transporter binding in binge eating women Psychopharmacology (Berl) 2001 14 310 314 10.1007/s002130100716 11432694

[B47] Tammela LI Rissanen A Kuikka JT Karuhnen LJ Bergstrøm KA Repo-Tihonen E Naukkarinen H Vanninen E Tiihonen Y Uusitupa M Treatment improves serotonin transporter binding and reduces binge eating Psychopharmacology (Berl) 2003 14 89 93 10.1007/s00213-003-1519-6 12768277

[B48] Koskela AK Kaurijoki S Pietiläinen KH Karhunen L Pesonen U Kuikka JT Kaprio J Rissanen A Serotonin transporter binding and acquired obesity - An imaging study of monozygotic twin pairs Physiol Behav 2008 14 724 732 10.1016/j.physbeh.2007.11.043 18177905

[B49] Matsumoto R Inverse correlation between body mass index and serotonin transporter in human brain: A [^11^C]DASB PET study Neuroimage 2008 14 Suppl 2 T161

[B50] Erritzoe D Frokjaer VG Haahr MT Kalbitzer J Svarer C Holst KK Hansen DL Jernigan TL Lehel S Knudsen GM Cerebral serotonin transporter binding is inversely related to body mass index Neuroimage 2010 14 284 289 10.1016/j.neuroimage.2010.03.086 20382236

[B51] Giannaccini G Betti L Palego L Schmid L Fabbrini L Pelosini C Gargini C Da Valle Y Lanza M Marsili A Maffei M Santini F Vitti P Pinchera A Lucacchini A Human Serotonin Transporter Expression during Megakaryocytic Differentiation of MEG-01 Cells Neurochem Res 2010 14 628 635 10.1007/s11064-009-0112-8 20041293

[B52] Giannaccini G Betti L Palego L Pirone A Schmid L Lanza M Fabbrini L Pelosini C Maffei M Santini F Pinchera A Lucacchini A Serotonin transporter (SERT) and translocator protein (TSPO) expression in the obese *ob/ob* mouse BMC Neurosci 2011 14 18 10.1186/1471-2202-12-18 21299850 PMC3044656

[B53] Charnay Y Cusin I Vallet PG Muzzin P Rohner-Jeanrenaud F Bouras C Intracerebroventricular infusion of leptin decreases serotonin transporter binding sites in the frontal cortex of the rat Neurosci Lett 2000 14 89 92 10.1016/S0304-3940(00)00951-4 10739882

[B54] Maffei M Halaas J Ravussin E Pratley RE Lee GH Zhang Y Fei H Kim S Lallone R Ranganathan S Leptin levels in human and rodent: measurement of plasma leptin and ob RNA in obese and weight-reduced subjects Nat Med 1995 14 1155 1161 10.1038/nm1195-1155 7584987

[B55] Considine RV Sinha MK Heiman ML Serum immunoreactive-leptin concentrations in normal-weight and obese humans N Engl J Med 1996 14 292 295 10.1056/NEJM199602013340503 8532024

[B56] Murphy DL Uhl GR Holmes A Ren-Patterson R Hall FS Sora I Detera-Wadleigh Lesch KP Experimental gene interaction studies with SERT mutant mice as models for human polygenic and epistatic traits and disorders Genes Brain Behav 2003 14 350 364 10.1046/j.1601-1848.2003.00049.x 14653307

[B57] Gardier AM Mutant mouse models and antidepressant drug research: focus on serotonin and brain derived neurotrophic factor Behavioral Pharmacol 2009 14 18 32 10.1097/FBP.0b013e3283243fcd 19179848

[B58] Matarese G La Cava A The intricate interface between immune system and metabolism Trends Immunol 2004 14 193 200 10.1016/j.it.2004.02.009 15039046

[B59] Kinoshita M Ono K Horie T Nagao K Nishi H Kuwabara Y Takanabe-Mori R Hasegawa K Kita T Kimura T Regulation of adipocyte differentiation by activation of serotonin (5-HT) receptors 5-HT_2AR_ and 5-HT_2CR_ and involvement of microRNA-448-mediated repression of KLF5 Mol Endocrinol 2010 14 1978 1987 10.1210/me.2010-0054 20719859 PMC5417392

[B60] Stunes AK Reseland JE Hauso O Kidd M Tømmerås K Waldum HL Syversen U Gustafsson BI Adipocytes express a functional system for serotonin synthesis, reuptake and receptor activation Diabetes Obes Metab 2011 14 551 558 10.1111/j.1463-1326.2011.01378.x 21320265

[B61] Breum L Rasmussen MH Hilsted J Fernstrom JD Twenty-four-hour- plasma tryptophan concentrations and ratios are below normal in obese subjects and are not normalized by substantial weight reduction Am J Clin Nutr 2003 14 1112 1118 12716660 10.1093/ajcn/77.5.1112

[B62] Hariri AR Holmes A Genetic of emotional regulation: the role of serotonin transporter in neural function Trends Cogn Sci 2006 14 182 191 10.1016/j.tics.2006.02.011 16530463

[B63] Park SY Harrold JA Widdowson PS Williams G Increased binding at 5-HT_1A_, 5-HT_1B_ and 5-HT_2A_ receptors and 5-HT transporter in diet-induced obese rats Brain Res 1999 14 90 97 10.1016/S0006-8993(99)02055-7 10564740

[B64] Tripp A Sibille E Kalueff AV, Laporte JL SERT models of emotional dysregulation Experimental Models in Serotonin Transporter Research 2009 Cambridge, UK: Cambridge University Press 105 135

[B65] Obesity: preventing and managing the global epidemic. Report of a WHO consultation World Health Organ Tech Rep Ser 2000 14 1 253 11234459

[B66] Mc Pherson GA Grant A Analysis of radioligand binding experiments, a collection of computer programs for IBM PC J Pharmacol Methods 1985 14 213 288 10.1016/0160-5402(85)90034-8 3840547

